# Xpp1 regulates the expression of xylanases, but not of cellulases in *Trichoderma reesei*

**DOI:** 10.1186/s13068-015-0298-8

**Published:** 2015-08-06

**Authors:** Christian Derntl, Alice Rassinger, Ewald Srebotnik, Robert L Mach, Astrid R Mach-Aigner

**Affiliations:** Department for Biotechnology and Microbiology, Institute of Chemical Engineering, TU Wien, Gumpendorfer Str. 1a, 1060 Vienna, Austria; Department of Biochemical Engineering, Institute of Chemical Engineering, TU Wien, Gumpendorfer Str. 1a, 1060 Vienna, Austria

**Keywords:** *Trichoderma reesei*, Xylanases, Cellulases, Gene regulation, Transcription factor, Cellulosic ethanol

## Abstract

**Background:**

The ascomycete *Trichoderma reesei* is industrially used for the production of cellulases. During the production process xylanases are co-secreted, which uses energy and nutrients. Cellulases and xylanases share the same main regulators, which makes a knowledge-based strain design difficult. However, previously a *cis*-element in the promoter of the main xylanase-encoding gene was identified as binding site for a putative repressor. Subsequently, three candidate repressors were identified in a pull-down approach. The expression of the most promising candidate, Xpp1 (Xylanase promoter-binding protein 1), was reported to be up-regulated on the repressing carbon source d-glucose and to bind the *cis*-element in vitro.

**Results:**

In this study, Xpp1 was deleted and over-expressed in *T. reesei*. An in vivo DNA-footprint assay indicated that Xpp1 binds a palindromic sequence in the *xyn2* promoter. Comparison of the deletion, the over-expression, and the parent strain demonstrated that Xpp1 regulates gene expression of xylanolytic enzymes at later cultivation stages. Xpp1 expression was found to be up-regulated, additionally to d-glucose, by high d-xylose availability. These findings together with the observed *xyn2* transcript levels during growth on xylan suggest that Xpp1 is the mediator of a feedback mechanism. Notably, Xpp1 has neither influence on the d-xylose metabolism nor on the expression of cellulases.

**Conclusions:**

Xpp1 as regulator acting on the expression of xylanases, but not cellulases, is a highly promising candidate for knowledge-based strain design to improve the cellulases-to-xylanases ratio during industrial cellulase production.

**Electronic supplementary material:**

The online version of this article (doi:10.1186/s13068-015-0298-8) contains supplementary material, which is available to authorized users.

## Background

The filamentous ascomycete *Trichoderma reesei* (teleomorph, *Hypocrea jecorina*) [1]) is industrially used for its outstanding secretory capacities. Industry strains produce over 100 g/L of enzyme in industrial-scale processes [2]. The main component of the secreted enzymes is the cellobiohydrolase CBHI (EC 3.2.1.91). Further components of the secreted enzyme cocktail are the cellobiohydrolase CBHII (EC 3.2.1.91), the endoglucanase EGLI (EC 3.2.1.4), and the *β*-glucosidase BGLI (EC 3.2.1.21) [3, 4]. These enzymes work synergistically to break down cellulose to d-glucose. *T. reesei* cellulases find use in a wide range of industrial applications, such as paper and pulp, textile, and food and feed industry [5], and are still a bottleneck for cost-effective production of cellulosic ethanol [6].

However, *T. reesei* secretes also other enzymes along with the latter mentioned cellulases—most prominently the major endo-*β*-1,4-xylanase XYNII (EC.3.2.1.8) [7] and the *β*-xylosidase BXLI (EC 3.2.1.37) [8]. Endo-*β*-1,4-xylanases cleave the backbone of xylan (i.e., the *β*-1,4-d-xylose chain) in the middle, generating substrates for the *β*-xylosidase which cleaves off d-xylose from the non-reducing ends of the xylan backbone. The co-secretion of cellulases and xylanases makes sense for the saprophyte *T. reesei*, because cellulose in plant material is encountered mostly exclusively together with hemicelluloses in the lignocellulose complex (reviewed in [9]). In industrial applications, however, the co-secretion of xylanases is a disadvantage for cost-efficient cellulase production because *T. reesei* uses additional energy and nutrients for their expression. Therefore, the reduction of xylanase formation during the cellulase production process is an obvious possibility for a more efficient cellulase production.

A way to achieve this would be knowledge-based strain design by deleting and/or over-expressing transcription factors that specifically regulate xylanase gene expression. However, the same main regulators regulate gene expression of cellulases and xylanases in *T. reesei*. The transactivator Xyr1 (Xylanase regulator 1) is essential for gene expression of most cellulolytic and xylanolytic enzymes [10]. Notably, cellulases seem to be regulated in a different manner by Xyr1 than xylanases. The up-regulation of *xyr1* transcription goes hand in hand with the up-regulation of gene expression of cellulolytic enzymes, whereas the regulation of xylanases seems to depend on additional mechanisms and factors [11]. Further, gene expression of cellulases, xylanases, and their main regulator Xyr1 is subjected to carbon catabolite repression (CCR) [12–14] mediated by the transcription factor Cre1 (Carbon catabolite repressor 1) [15]. CCR is triggered by high concentrations of d-glucose and d-xylose (products of enzymatic degradation of cellulose and xylan, respectively) [12–14, 16]. Additionally, all further described transcription factors involved in the regulation of xylanases, i.e., Ace1, Ace2, and Ace3, were reported to have also effects on the gene expression of cellulases [13, 17–20]. Thus, none of the so far known regulators can be specifically used as a target for knowledge-based strain design with the aim of shifting the cellulases-to-xylanases ratio.

In 2003, a *cis*-element in the *xyn2* promoter bound under repressing conditions was identified. It was described as an AGAA sequence on the non-coding strand upstream of an Xyr1 binding site [21]. A mutation of the AGAA-box led to an increased expression of a reporter gene under inducing conditions. The AGAA-box was therefore considered to be bound by a repressor. More recently, based on the development of a highly sensitive in vivo footprinting technique, an inverted repeat of the AGAA sequence further upstream was suggested to be part of the binding motif for the potential repressor [22].

Three candidate regulators binding the *cis*-element were identified by a pull-down assay followed by mass-spectrometric analysis and bioinformatic assessment [23]. The expression of the most promising regulatory protein, Xpp1 (Xylanase promoter-binding protein 1), was reported to be up-regulated in the presence of d-glucose. Additionally, a GST fusion of its DNA-binding domain as well as an in vitro translated full-length protein could bind a DNA probe containing the *cis*-element of the *xyn2* promoter in vitro [23].

In this study, we tested the influence of different carbon sources on the level of *xpp1* expression, and deleted and over-expressed *xpp1* in *T. reesei*. To investigate the involvement of Xpp1 in the regulation of xylanase and cellulase expression in vivo, the resulting strains were compared with regard to their xylanolytic and cellulolytic activities by enzyme assays and by direct measurement of the degradation compounds. Transcript levels of the genes encoding for the main enzymes involved in the degradation of xylan and cellulose were determined. Moreover, binding of Xpp1 in vivo to its *cis*-element in the *xyn2* promoter was investigated by in vivo footprinting.

## Results

### Deletion and over-expression of *xpp1* in *T. reesei*

To analyze the role of Xpp1 in vivo, we deleted and over-expressed *xpp1* in *T. reesei*. For the deletion of *xpp1*, a gene replacement strategy by homologous recombination was applied. The structural gene of *xpp1* was replaced by a hygromycin resistance cassette in *T. reesei* QM6aΔ*tmus53*. A schematic representation of the deletion strategy is shown in Additional file 1a. For over-expression of *xpp1*, a co-transformation strategy was applied. The coding sequence of *xpp1* fused to the constitutive promoter of *pki* was inserted ectopically into the chromosome of *T. reesei* QM6a using pAN7-1, which confers hygromycin resistance [24]. The transcript levels of *xpp1* were measured in the obtained candidates by quantitative PCR (qPCR). A deletion strain lacking *xpp1* transcript was chosen randomly and termed QM6aΔ*xpp1*. The over-expression candidate exhibiting the highest relative expression of *xpp1* compared to its parent strain (3.5-fold over-expressed) was chosen for further experiments and termed QM6aOE*xpp1*. Genomic modifications in both strains were confirmed by PCR and by Southern blot analysis (Additional file 1b, c). Neither the deletion strain QM6aΔ*xpp1* nor the over-expression strain QM6aOE*xpp1* showed any differences in growth or sporulation behavior compared to their parent strains in minimal medium (liquid cultures or plates) containing lactose or carboxymethylcellulose (CMC), and surprisingly neither on xylan. On d-glucose, the deletion strain grows slightly slower compared to the parent strain (see Additional file 2).

### In vivo DNA binding of Xpp1 under repressing conditions

**Fig. 1:**
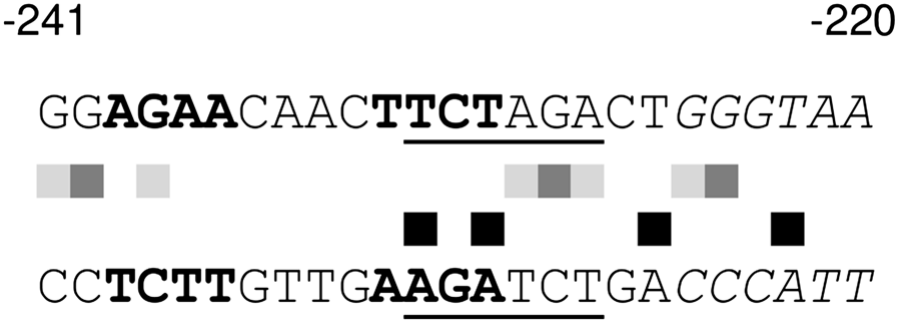
In vivo footprinting analysis of the Xpp1-binding region within the *xyn2* promoter. *T. reesei* QM6aΔ*tmus53* and the *xpp1* deletion strain were pre-cultured on glycerol and thereafter transferred to MA medium containing 50 mM d-glucose. DNA was methylated in vivo with DMS after 3 h incubation. Analysis of data was performed using ivFAST [22]. Significant differences in methylation intensities of individual purine nucleotides between the two strains are represented by *squares* (*white*, no difference; *light gray*, difference with ratios of more than 1.1 and less than 1.3; *dark gray*, differences with ratios of more than 1.3 and less 1.5; *black*, differences with ratios of more than 1.5). The sequence of the coding (*upper lane*) and non-coding (*lower lane*) strand from positions −241 to −220 of the *xyn2* promoter is given. *Bold letters* indicate the previously described AGAA-boxes, *underlined letters* the palindromic sequence, and *italic letters* an atypical Xyr1-binding site [20].

As mentioned, Xpp1 was identified by a pull-down assay using a probe that contained the AGAA-boxes of the *xyn2* promoter [23]. To test whether this motif is bound in vivo by Xpp1, we performed a dimethyl sulfate (DMS)-induced in vivo footprint comparing the *xpp1* deletion strain and its parent strain. In a replacement experiment, both strains were pre-grown on glycerol and then transferred to minimal medium containing 50 mM d-glucose. We could observe drastic differences between the two strains comparing the accessibility of the part of the *xyn2* promoter that contains the AGAA-boxes (Fig. 1). Two nucleotides in the downstream AGAA-box were strongly hypermethylated in the *xpp1* deletion strain, but only one nucleotide of the upstream AGAA-box. Interestingly, three nucleotides adjacent to the downstream AGAA-box were also hypermethylated. These three nucleotides are part of a palindromic sequence (5′-TCTAGA-3′) that overlaps with the downstream AGAA-box (Fig. 1).

### Induction of *xpp1* gene expression

**Fig. 2:**
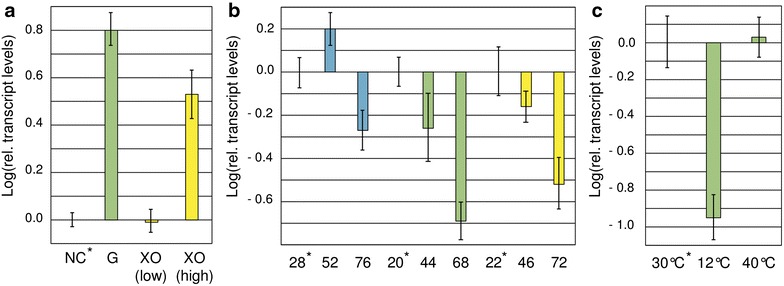
Gene expression of *xpp1.* **a** *T. reesei* QM6aΔ*tmus53* was pre-cultured on glycerol and thereafter incubated in MA media without carbon source (NC) or containing 50 mM d-glucose (G), or 0.5 mM d-xylose [XO (low)], or 66 mM d-xylose [XO (high)] for 3 h. **b** *T. reesei* QM6aΔ*tmus53* was grown in MA media containing 1% (w/v) glycerol (*blue bars*), d-glucose (*green bars*), or d-xylose (*yellow bars*) for indicated time periods (given in hours). **c** *T. reesei* QM6aΔ*tmus53* was pre-cultured on d-glucose at 30°C and thereafter incubated at indicated temperatures for 3 h. Transcript levels of *xpp1* were measured by qPCR using *sar1* and *act* transcript levels for normalization and were referred to the reference samples (indicated by *asterisks*). Results are given as relative transcript ratios in logarithmic scale (Log). The values provided in the figures are means from three biological experiments. *Error bars* indicate standard deviations.

Previously, the gene expression of *xpp1* was reported to be up-regulated by d-glucose [23]. We were interested whether d-xylose has an effect on the transcript levels of *xpp1*. To test this, *T. reesei* QM6aΔ*tmus53* was replaced on 50 mM d-glucose, 0.5 mM d-xylose, or 66 mM d-xylose, and transcript levels of *xpp1* were measured by a qPCR assay. We found both d-glucose and high levels of d-xylose to induce the gene expression of *xpp1* (Fig. 2a). To gain further insights into the regulation of gene expression of *xpp1*, we additionally compared its transcript levels in a growth experiment on different carbon sources, i.e., d-glucose, d-xylose, and glycerol [1% (w/v) each]. Samples were taken when similar amounts of biomass had accumulated on the different carbon sources. We found that transcript levels of *xpp1* decreased on all three carbon sources at the later time point (Fig. 2b). This finding prompted us to investigate whether this decrease was caused by the depletion of an easily utilizable carbon source or by a change in growth rate. To this end, we grew *T. reesei* in 1% (w/v) d-glucose for 24 h at 30°C and then changed the temperature for 3 h. We found the *xpp1* transcript levels drastically down-regulated at 12°C (Fig. 2c). On the other hand, a temperature shift to 40°C did not influence the *xpp1* transcript levels, indicating that eventual stress caused by the temperature change was not the reason for the observed down-regulation at 12°C.

### Deletion of *xpp1* leads to higher xylanolytic activities in *T. reesei*

**Fig. 3:**
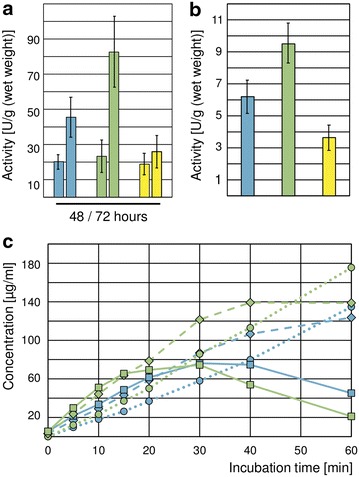
Influence of Xpp1 on the xylanolytic activities of *T. reesei. T. reesei* QM6aΔ*tmus53* (*blue bars*), the *xpp1* deletion strain (*green bars*), and the *xpp1* over-expression strain (*yellow bars*) were grown in MA medium containing 1% xylan for 48 and 72 h. The endo-xylanolytic activities (**a**) and the β-xylosidase activity (**b**) were measured and normalized to the acquired biomass after 72 h. The values provided in the figures are means from three biological experiments. *Error bars* indicate standard deviations. **c** Xylan was degraded in vitro with culture supernatants (xylan, 72 h) of *T. reesei* QM6aΔ*tmus53* (*blue*) and the *xpp1* deletion strain (*green*) in a time course experiment. Concentrations of xylopentaose (*squares*, *solid lines*), xylotriose (*diamonds*, *dashed lines*), and d-xylose (*circles*, *dotted lines*) are given.

To examine the influence of Xpp1—the suggested repressor of the expression of the endo-xylanase XYNII [21, 23]—on the xylanolytic phenotype of *T. reesei*, the *xpp1* deletion, over-expression, and parent strain were grown on xylan. We measured substantial higher endo-xylanolytic activity in the resulting culture supernatant of the deletion strain and lower endo-xylanolytic activity in the over-expression strain compared to the parent strain after 72 h (Fig. 3a). Additionally, we detected higher *β*-xylosidase activity in the culture supernatant of the deletion strain compared to its parent strain and lower activity in the over-expression strain (Fig. 3b).

We were interested whether the synergistic action of the xylanolytic enzymes differed between the deletion strain and its parent strain during cultivation on xylan. As we could not detect free oligo- or monosaccharides in the supernatant during the growth experiment, we used sterile-filtered culture supernatants of the two strains to degrade xylan in vitro. The degradation was monitored over time by measuring the concentration of free xylose oligomers and d-xylose. Xylose oligomers (xylobiose to xyloheptaose) were released at a higher initial rate in the assay using culture supernatant of the *xpp1* deletion strain compared to the one of the parent strain. The concentrations of xylopentaose and xylotriose are shown as examples in Fig. 3c. This points to a higher endo-xylanolytic activity of the *xpp1* deletion strain. However, the maximal intermediate concentration of xylopentaose did not increase for *xpp1* deletion, but was similar to that of the parent strain (approximately, 75 µg/mL; see Fig. 3c). Considering the higher endo-xylanolytic activity of the *xpp1* deletion strain, the observed similar concentration maxima additionally point to a higher *β*-xylanolytic activity because xylopentaose is primarily generated by the action of the endo-xylanases and degraded by *β*-xylosidase. This conclusion is supported by the faster increase of d-xylose concentration in the assay using culture supernatant of the *xpp1* deletion strain compared to the parent strain (Fig. 3c), since d-xylose is exclusively released by the action of *β*-xylosidase.

### Xpp1 regulates *xyn2* transcription at later cultivation stages

**Fig. 4:**
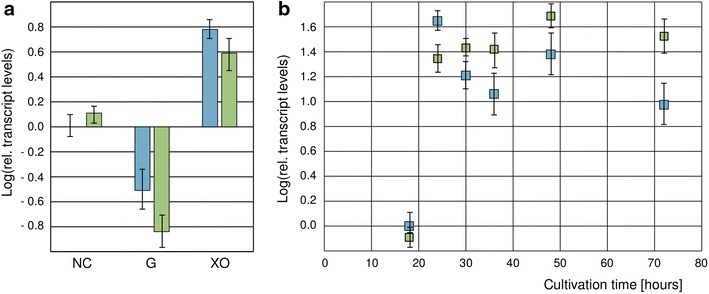
Influence of Xpp1 on transcript levels of *xyn2* in *T. reesei.* **a** *T. reesei* QM6aΔ*tmus53* (*blue bars*) and the *xpp1* deletion strain (*green bars*) were pre-cultured on glycerol and thereafter transferred to MA media without carbon source (NC), or containing 50 mM d-glucose (G), or 66 mM d-xylose (XO) and incubated for 3 h. Transcript levels refer to the reference sample (QM6aΔ*tmus53*, NC). **b** *T. reesei* QM6aΔ*tmus53* (*blue squares*) and the *xpp1* deletion strain (*green squares*) were grown in MA medium containing 1% (w/v) xylan. Samples were taken after 18, 24, 30, 36, 48, and 72 h of cultivation. Transcript levels of *xyn2* were measured by qPCR using *sar1* and *act* transcript levels for normalization and referred to the reference sample (QM6aΔ*tmus53*, 18 h). Results are given as relative transcript ratios in logarithmic scale (Log). The values provided in the figures are means from three biological experiments. *Error bars* indicate standard deviations.

We aimed to test whether Xpp1 does in fact act as a transcription factor on the regulation of gene expression of *xyn2* directly. In a first experiment, the *xpp1* deletion strain and its parent strain were subjected to a replacement experiment using 50 mM d-glucose or 66 mM d-xylose. The transcript levels of *xyn2* were quantified by a qPCR assay. Surprisingly, we could not observe higher *xyn2* transcript levels in the deletion strain on both tested carbon sources after 3 h compared to the parent strain (Fig. 4a). Xpp1 does not seem to have an effect on the inducibility of *xyn2* gene expression, which conflicts with the elevated xylanolytic activity of the *xpp1* deletion strain observed, e.g., after 72 h growth on xylan. Therefore, we monitored *xyn2* transcript levels in the two strains throughout growth on xylan. We could detect higher *xyn2* transcript levels in the deletion strain compared to its parent strain after 30, 36, 48, and 72 h (Fig. 4b). Only the early (24 h) *xyn2* transcript level is lower in the deletion strain. These findings match the observed xylanolytic activities. Deletion and parent strain had the same xylanolytic activities after 48 h (Fig. 3a). During growth *xyn2* transcript levels drop in the parent strain, whereas they stay at a high level in the deletion strain (Fig. 4b), which results in a higher xylanolytic activity of the deletion strain at later cultivation stages (Fig. 3a).

### Xpp1 controls the gene expression of *xyn1* and the putative *bxl2*

**Fig. 5:**
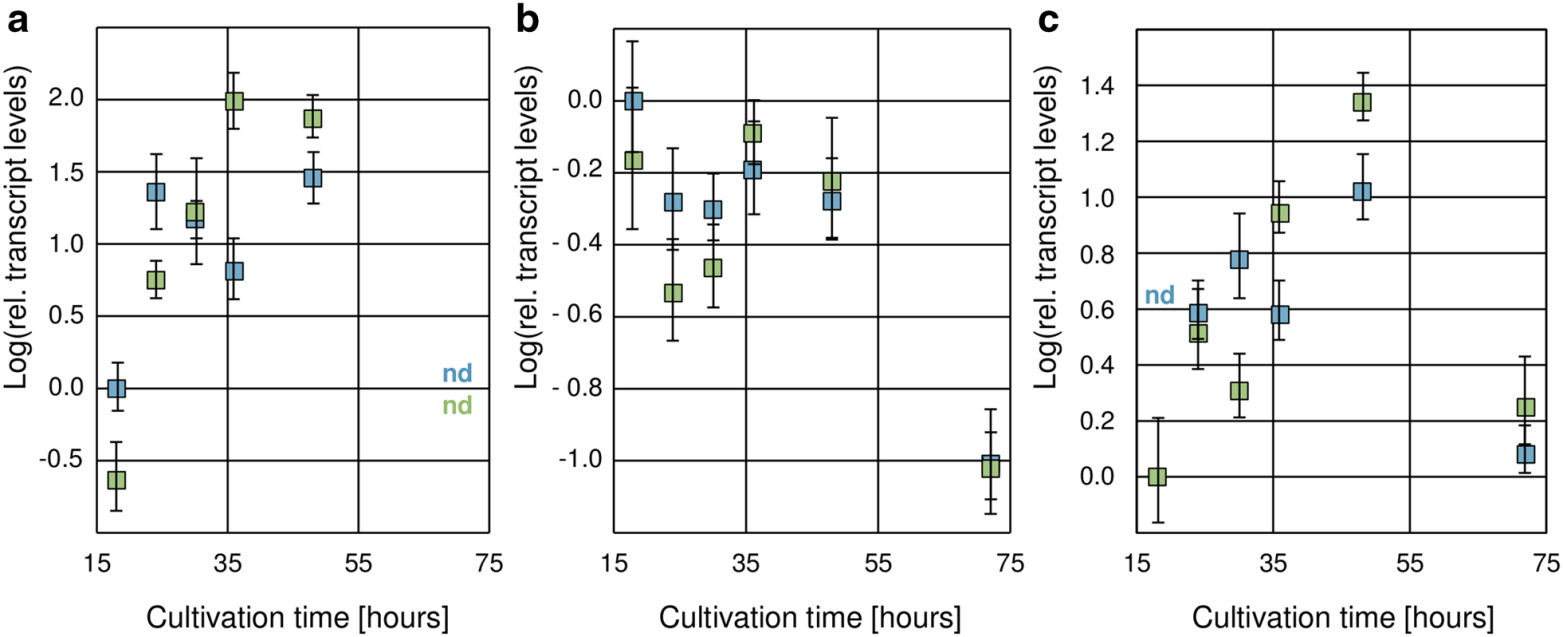
Influence of Xpp1 on transcript levels of *xyn1*, *bxl1*, and *bxl2* in *T. reesei. T. reesei* QM6aΔ*tmus53* (*blue squares*) and the *xpp1* deletion strain (*green squares*) were grown in MA medium containing 1% (w/v) xylan. Samples were taken after 18, 24, 30, 36, 48, and 72 h growth. Transcript levels of *xyn1* (**a**), *bxl1* (**b**), and *bxl2* (**c**) were measured by qPCR using *sar1* and *act* transcript levels for normalization and referred to the reference sample (*T. reesei* QM6aΔ*tmus53*, 24 h for *xyn1* and *bxl1*; *xpp1* deletion strain, 24 h for *bxl2*). Results are given as relative transcript ratios in logarithmic scale (Log). The values provided in the figures are means from three biological experiments. *Error bars* indicate standard deviations. *nd* not detected.

Since further enzymes besides XYNII contribute to the xylanolytic activities of *T. reesei,* we investigated if Xpp1 also controls their expression. First, we analyzed *xyn1* transcript levels, which turned out to be higher in the *xpp1* deletion strain compared to the parent strain (Fig. 5a), similar to *xyn2* (compare Fig. 4b). Since we detected increased *β*-xylosidase activity in the supernatant of the *xpp1* deletion strain (compare Fig. 3b), we measured the *bxl1* transcript levels. Surprisingly, we detected equal levels in both strains (Fig. 5b), which contradicts the measured enzyme activity. However, the protein ID 58450 (on http://genome.jgi-psf.org/Trire2/Trire2.home.html) is annotated as a putative *β*-xylosidase [25]. Consequently, we analyzed the transcript levels of the gene encoding for this candidate *β*-xylosidase (in the following termed *bxl2*). They were indeed higher in the *xpp1* deletion strain compared to its parental strain at later cultivation stages (Fig. 5c).

### Xpp1 does not affect the initial step of d-xylose metabolism

**Fig. 6:**
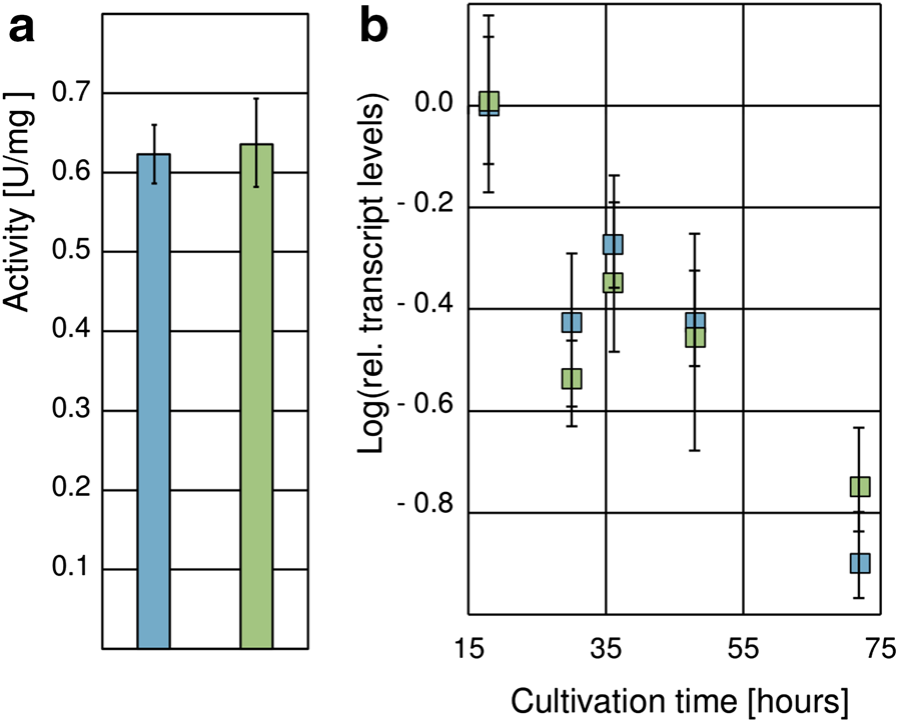
Influence of Xpp1 on xylose reductase expression in *T. reesei. T. reesei* QM6aΔ*tmus53* (*blue*) and the *xpp1* deletion strain (*green*) were grown in MA medium containing 1% (w/v) xylan. Samples were taken after 18, 30, 36, 48, and 72 h growth. **a** Xylose reductase activity in cell-free extracts was measured in vitro and normalized to the total protein concentration of the cell-free extracts. **b** Transcript levels of the *xyl1* gene were measured by qPCR using *sar1* and *act* transcript levels for normalization and were referred to the reference sample (*T. reesei* QM6aΔ*tmus53*, 18 h). Results are given as relative transcript ratios in logarithmic scale (Log). The values provided in the figures are means from three biological experiments. *Error bars* indicate standard deviations.

Degradation of xylan results in the release of d-xylose, which is taken up by *T. reesei* and metabolized via the pentose phosphate way. The initial reaction is the reduction of d-xylose to xylitol, catalyzed by xylose reductase (EC1.1.1.307). We measured xylose reductase activity of the *xpp1* deletion strain and its parent strain after 72 h growth on xylan. We could not detect any difference between the two strains (Fig. 6a). Further, the transcript levels of *xyl1*, encoding the xylose reductase, were monitored in the two strains throughout growth on xylan. Samples were taken periodically and *xyl1* transcript levels were measured with a qPCR assay. Matching the obtained enzymatic activities, no differences of *xyl1* transcript levels could be observed between the two strains (Fig. 6b). Xpp1 regulates the gene expression of the extracellular xylanolytic enzymes, but not of the intracellular xylose reductase. We presume that the xylose reductase can be used as an indicator for the whole downstream d-xylose metabolism in this context.

### Cellulase expression is not regulated by Xpp1

**Fig. 7:**
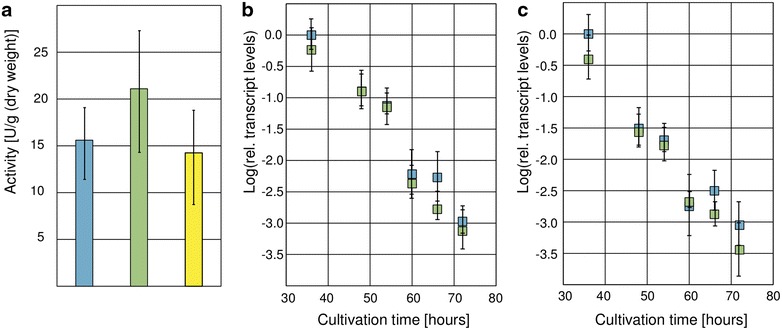
Influence of Xpp1 on cellulase expression in *T. reesei. T. reesei* QM6aΔ*tmus53* (*blue*), the *xpp1* deletion strain (*green*), and the *xpp1* over-expression strain (*yellow*) were grown in MA medium containing 1% (w/v) CMC for 36, 48, 54, 60, 66, and 72 h. **a** Endo-cellulolytic activity was measured and normalized to the acquired biomass after 72 h. Relative transcript levels of *cbh1* (**b**) and *egl1* (**c**) were measured by qPCR using *sar1* and *act* transcript levels for normalization and were referred to the reference sample (*T. reesei* QM6aΔ*tmus53*, 36 h). Results are given as relative transcript ratios in logarithmic scale (Log). The values provided in the figures are means from three biological experiments. *Error bars* indicate standard deviations.

Owing to the fact that cellulolytic and xylanolytic enzymes are co-regulated in *T. reesei*, we were interested to know to what extent Xpp1 influences the expression of cellulases. The *xpp1* deletion strain, the over-expression strain, and the parent strain were cultivated in minimal medium containing CMC for 72 h, and endo-cellulolytic activities of the resulting culture supernatants were assayed. We could not detect any differences on comparing the three strains (Fig. 7a). Further, transcript levels of the main cellulases, i.e., *cbh1*, *cbh2*, and *egl1*, were monitored throughout growth in CMC with qPCR assays. No differences between the deletion strain and the parent strain could be observed (Fig. 7b, c, data for *cbh2* not shown).

## Discussion

Two inverted AGAA-boxes in the *xyn2* promoter were previously considered to be the binding motif of a repressor [21, 22]. Xpp1 was later identified as the most promising candidate repressor [23]. Using in vivo footprinting, we could detect strong differences at the putative binding site of Xpp1 in the *xyn2* promoter comparing the *xpp1* deletion strain and its parent strain. However, Xpp1 is a basic helix-loop-helix protein containing a predicted E-box binding motif [23]. The classical E-box is a hexameric palindrome with the consensus 5′-CANNTG-3′ [26]. Interestingly, the AGAA-box on the non-coding strand within the *xyn2* promoter overlaps with a hexameric palindrome (5′-TCTAGA-3′). Five of the six nucleotides of this palindromic sequence were hypermethylated during in vivo footprinting in the absence of Xpp1. A similar hexameric palindrome (5′-ACTAGT-3′) can be found in the promoters of *xyn1* and *bxl2*. Notably, both promoters contain also an inverted AGAA-repeat. In summary, we observed a correlation between regulation by Xpp1 and the presence of a hexameric palindrome 5′-WCTAGW-3′ together with an inverted AGAA-repeat. Table 1 provides an overview on the genes investigated during this study as potential targets of Xpp1 regulation and the presence of relevant *cis* elements in their promoters.

**Table 1:** Overview on investigated genes concerning the Xpp1 regulation

Gene	Regulated by Xpp1	Inverted AGAA repeat^a^	WCTAGW palindrom^a^
*xyn2*	X	X	X
*xyn1*	X	X	X
*bxl1*	–	–	–
*bxl2*	X	X	X
*xyl1*	–	–	–
*cbh1*	–	–	–
*cbh2*	–	X	–
*egl1*	–	–	–
*xyr1*	–	–	–

^a^Present in the promoter within 1 kbp upstream of the start codon.

Further, an atypical Xyr1 binding site [20] downstream of the palindromic sequence within the *xyn2* promoter was also hypermethylated in the in vivo footprint assay in the *xpp1* deletion strain, notably, to a lesser extent than the palindromic motif. However, we could not observe a difference of *xyr1* transcript levels comparing deletion and parent strain during growth on xylan (unpublished observations by Derntl C, Mach RL, Mach-Aigner AR). The observed differences might be the mere result of a changed accessibility of the whole region due to the absence of Xpp1.

Deletion of *xpp1* resulted in elevated *xyn1*, *xyn2,* and *bxl2* transcript levels at later cultivation stages in the deletion strain and in higher endo-xylanolytic and *β*-xylosidase activities in the supernatant after 72 h, but not after 48 h. Xpp1 seems to act as a repressor of gene expression of xylanolytic enzymes at later time points. Transcript levels of *xyn2*, *xyn1*, and *bxl2* are higher in the deletion strain compared to the parent strain at late cultivation stages. We speculate that these observed differences are the result of a CCR-independent feedback mechanism mediated by Xpp1. During this study, we observed that high concentrations of d-xylose up-regulate Xpp1 expression, which acts as repressor on the expression of xylanolytic enzymes. Considering that accumulation of high amounts of d-xylose is a relatively slow process, this would fit the general idea of a feedback mechanism. This model would also explain why Xpp1 does not influence the inducibility of *xyn2* expression at early time points (compare Fig. 4a, b). According to our hypothesis, the role of Xpp1 is the down-regulation of an induced system, but not the prevention of induction. The nature of this feedback mechanism might also explain the observed equal growth behavior of parent, deletion, and over-expression strains. All strains have similar initial xylanolytic activities and therefore provide similar amounts of monosaccharides for the growing fungi in the beginning. Biomass formation occurs during this period, resulting in similar growth behaviors.

Remarkably, Xpp1 regulates only the xylanolytic part of the lignocellulose-degrading enzymes in *T. reesei*. Transcript levels of the main cellulases as well as cellulolytic activities were not changed in the deletion and the over-expression strain compared to the parental strain. As mentioned earlier, regulation of gene expression of the main cellulases seems to be regulated in a direct manner by the amount of available Xyr1 [11]. Matching the obtained results, we could not observe a difference of *xyr1* transcript levels comparing deletion and parent strain throughout growth on CMC (unpublished observations by Derntl C, Mach RL, Mach-Aigner AR).

## Conclusions

Xpp1 seems to be the mediator of a feedback mechanism regulating gene expression of xylanases. Xpp1 does not influence transcription of the main cellulolytic enzymes. We believe that this makes Xpp1 a highly attractive candidate for knowledge-based strain design, as this regulatory protein is a very promising instrument for shifting the cellulases-to-xylanases ratio in the secreted protein fraction during industrial cellulase production.

## Methods

### Fungal strains

*T. reesei* strains QM6a (ATCC 13631), QM6aΔ*tmus53* [27], the *xpp1* deletion strain QM6aΔ*tmus53*Δ*xpp1* (QM6aΔ*xpp1,* this study), and the *xpp1* over-expression strain (QM6aOE*xpp1*, this study) were maintained on malt extract (MEX) agar at 30°C. Hygromycin B was added when applicable to a final concentration of 113 U/mL.

### Growth conditions

For carbon source replacement experiments, strains were pre-cultured in 1-L Erlenmeyer flasks on a rotary shaker (180 rpm) at 30°C for 22 h in 300 mL of Mandels–Andreotti (MA) medium [28] containing 1% (w/v) glycerol as the sole carbon source. A total of 10^9^ conidia per liter (final concentration) was used as the inoculum. Pre-grown mycelia were washed, then equal amounts were resuspended in MA media containing d-xylose and d-glucose in final concentrations as given or in medium without carbon source (reference condition), and harvested after 3 h of incubation.

For direct cultivation on d-glucose, d-xylose, glycerol, or xylan (Lenzing AG, Lenzing, Austria) strains were grown in 500-mL Erlenmeyer flasks in a rotary shaker (180 rpm) at 30°C in 100 mL of MA medium containing 1% (w/v) of the respective carbon source. For direct cultivation on CMC, strains were grown in 1-L Erlenmeyer flasks stationary at 30°C in 60 mL of MA medium containing 1% (w/v) CMC (Carl Roth GmbH, Karlsruhe, Germany). Mycelium and supernatant were separated by filtration through Miracloth (EMD Millipore, part of Merck KGaA, Darmstadt, Germany). Mycelia grown on xylan were weighed directly as reference for enzymatic assays. Mycelia grown on CMC were dried at 80°C overnight prior to weighing.

### Plasmid construction

*Escherichia coli* strain Top10 (Invitrogen, part of Life Technologies, Paisley, UK) was used for all cloning purposes throughout this study and maintained on LB at 37°C. Ampicillin and hygromycin B were added when applicable to final concentrations of 100 mg/mL and 113 U/mL, respectively.

PCRs for all cloning purposes were performed with Phusion High-Fidelity DNA Polymerase (Thermo Scientific, Waltham, MA, USA) according to the manufacturer’s instructions. All used primers are listed in Additional file 3. Generation of competent *E. coli* cells and subsequent transformation was performed according to standard protocols using CaCl_2_.

For the construction of pCD-Δxpp1, the 5′-flank of *xpp1* was amplified by PCR using chromosomal DNA of *T. reesei* QM6a as template with the primers xpp1-5fwD-NotI and xpp1-5rev. The PCR product was inserted into an EcoRV-digested pJET1.2 (Thermo Scientific) in the opposite direction of *Eco47IR* (killer gene), yielding pJET-5′-xpp1. The 3′-flank of *xpp1* was amplified by PCR using chromosomal DNA of *T. reesei* QM6a as template with the primers xpp1-3fwd and xpp1-3rev-NotI. This PCR product was inserted into pJET-5′-xpp1 in the same direction as the 5′-flank. For this purpose, the plasmid was digested with ClaI and blunted with T4 DNA polymerase. The resulting plasmid was termed pJET-BF*xpp1*. Finally, a hygromycin resistance cassette was generated by PCR using pRLM_EX_30 [29] as template with the primers Ppki_5fwd and Tcbh2_rev-BcuI and inserted into pJET-BF*xpp1* which was previously digested with NcoI and blunted with T4 DNA polymerase. The orientation was determined by sequencing and found to be the same as the 5′-flank and the 3′-flank of *xpp1*.

For construction of pCD_ex_xpp1, the coding sequence of *xpp1* was amplified by PCR using cDNA from *T. reesei* QM6a as template with the primers xpp1_fwD-XbaI and xpp1_rev-NsiI. The PCR product was digested with XbaI and NsiI and ligated into pRLM_EX_30 [29] digested accordingly.

### Fungal protoplast transformation

Protoplast transformation of *T. reesei* was performed as described by Gruber et al. [30]. For deletion of *xpp1*, NotI-digested pCD-Δxpp1 was used for transformation of *T. reesei* QM6aΔ*tmus53*. For over-expression of *xpp1* under the control of the constitutive promoter of *pki*, pCD_ex_xpp1 was co-transformed together with pAN7-1 [24] into *T. reesei* QM6a. The transformation reaction was added to 80 mL melted, 50°C warm MEX agar containing 1.2 M sorbitol. This mixture was poured into four sterile Petri dishes and incubated at 30°C for 2–5 days until colonies were visible. The resulting candidates were subjected to four rounds of homokaryon selection by streaking.

### Isolation of chromosomal DNA and PCR analysis

Chromosomal DNA was isolated from mycelium by grinding in liquid nitrogen followed by a phenol/chloroform extraction. RNA was degraded using RNaseA (Thermo Scientific). DNA was precipitated with isopropanol, washed with 70% (v/v) ethanol, and dissolved in ddH_2_O. For PCR analysis, 10 ng of chromosomal DNA was used as template in a 25-µL PCR using GoTaq^®^ G2 polymerase (Promega, Madison, WI, USA) according to the manufacturer’s instructions. All used primers are listed in Additional file 3. For subsequent agarose gel electrophoresis of DNA fragments, a GeneRuler 1 kb DNA Ladder (Thermo Scientific) was applied for estimation of fragment size. DNA sequencing was performed at Microsynth (Balgach, Switzerland).

### Southern blot analysis

15 µg of chromosomal DNA were digested with 30 U of the given restriction enzymes. The resulting DNA fragments were separated by electrophoresis on a 0.8% agarose gel, then denatured in 0.4 M NaOH, and transferred by capillary forces onto a Biodyne B 0.45 µm nylon membrane (Pall Corporation, Port Washington, NY, USA) using 10 × SSC. 1.5 µg of biotinylated DNA probe were used for hybridization at 65°C overnight. Labeling of the probe was performed using a Klenow Fragment (exo-) (Thermo Scientific), random hexamer primers, and biotin-11-dUTP (Jena Bioscience, Jena, Germany). Signals were visualized by using Poly-HRP conjugated to streptavidin and ECL Plus Western Blotting substrate (both Thermo Scientific Pierce, part of Life Technologies) on a ChemiDoc MP (Bio-Rad Laboratories, Hercules, USA).

### RNA extraction and reverse transcription

0.01–0.03 g of harvested mycelia were homogenized in 1 mL of peqGOLD TriFast DNA/RNA/protein purification system reagent (PEQLAB Biotechnologie, Erlangen, Germany) using a FastPrep FP120 BIO101 ThermoSavant cell disrupter (Qbiogene, Carlsbad, USA). RNA was isolated according to the manufacturer’s instructions, and the concentration was measured using the NanoDrop 1000 (Thermo Scientific).

Synthesis of cDNA from mRNA was carried out using the RevertAid™ H Minus First Strand cDNA Synthesis Kit (Thermo Scientific) according to the manufacturer’s instructions.

### Transcript analysis

Quantitative PCRs were performed in a Mastercycler^®^ ep realplex 2.2 system (Eppendorf, Hamburg, Germany). All reactions were performed in triplicate. The amplification mixture (final volume 25 μL) contained 12.5 μL 2×  iQ SYBR Green Mix (Bio-Rad Laboratories, Hercules, USA), 100 nM forward and reverse primer, and 2.5 μL cDNA (diluted 1:100). Primer sequences are provided in Additional file 3. Cycling conditions and control reactions were performed as described previously [31]. Calculations using *sar1* and *act1* as reference genes were performed as published previously [31].

### In vivo footprinting

In vivo methylation of DNA using DMS followed by ligation-mediated PCR was performed as described previously [22] using the primers RG127, RG128, RG129, RG130, RG131, and RG132 for investigation of a regulatory region bearing the AGAA-box within the *xyn2* promoter. Primer sequences are provided in Additional file 3. FAM-labeled fragments were analyzed by capillary gel electrophoresis (Microsynth) and results were analyzed using the program ivFAST [22].

### Determination of enzymatic activities

Endo-xylanolytic and endo-cellulolytic activities of cultivation supernatants were measured with Xylazyme AX tablet assay and Cellazyme C tablet assay (both Megazyme International Ireland, Wicklow, Ireland) according to the manufacturer’s instructions, respectively. One unit of activity is defined as the amount of enzyme required to release 1 μmole of reducing-sugar equivalents per minute.

The *β*-xylosidase activity was determined with the substrate 4-nitrophenyl-*β*-d-xylopyranoside (Sigma-Aldrich Corporation, St. Louis, MO, USA) as described previously [32]. One unit of activity is defined as the amount of enzyme required to release 1 µmol of d-xylose reducing-sugar equivalents per minute.

For the measurement of xylose reductase activity, mycelia were ground in liquid nitrogen with mortar and pestle. 100 mg was resuspended in 1 mL 0.1 M Tris/HCl pH 7.5, 1 mM EDTA, and 5 mM *β*-mercaptoethanol and incubated at 4°C for 15 min. Cell debris was removed by centrifugation at 20,000*g* at 4°C for 10 min. The protein concentration of the resulting cell extract was determined photometrically by measuring the absorption at 280 nm using NanoDrop 1000 (Thermo Scientific). 200 µL of the cell extract was added to a 2 mL reaction in 0.1 M Tris, pH 7.5, containing 10 mM d-xylose and 0.15 mM NADPH. Oxidation of NADPH to NAD+ was monitored photometrically at 340 nm in a Jasco V-360 spectrophotometer (Jasco Corporation, Hachioji City, Japan) for 1 min at 25°C. One unit is defined as the amount that causes a reduction of optical density at 340 nm of 0.01 per minute. The activity was normalized to the total protein concentration of the cell extract.

### High-performance anion exchange chromatography with pulsed amperometric detection (HPAEC-PAD) analysis

For monitoring xylan degradation in vitro, 1 mL sterile-filtered cultivation supernatant from 72 h growth in MA medium containing 1% xylan (Lenzing AG) was mixed with 9 mL sterile MA medium containing 1% xylan (Lenzing AG) and incubated in a 50 mL sterile reaction tube on a rotary shaker (180 rpm) at 30°C. 200 µL samples were drawn at given time points and the enzymatic reactions immediately stopped by addition of 200 µL 100 mM NaOH. The remaining, undissolved xylan was removed by centrifugation at 4°C and 20,000*g* for 10 min. Samples were diluted 1:10 with 10 mM NaOH for HPAEC-PAD analysis. HPAEC-PAD analysis was performed with a Thermo Scientific Dionex ICS-5000 system (Thermo Scientific). Samples were separated on a Dionex CarboPac PA1 (2 × 250 mm) carbohydrate column (Thermo Scientific). The column was preconditioned at 0.26 mL/min in 150 mM NaOH/250 mM NaOAc for 5 min, followed by 150 mM NaOH for 15 min. Samples were then injected and oligosaccharides separated by a linear gradient to 150 mM NaOH/250 mM NaOAc in 20 min. Quantification was done by calibration of peak areas with authentic standards of xylooligosaccharides (Megazyme International, Ireland).

## Additional files

Additional file 1: Deletion and over-expression of *xpp1* (A) Deletion of *xpp1* in the parent strain QM6aΔ*tmus53* (Δ*tmus53*) by homologous recombination with the plasmid pCD-Δxpp1 yielding an *xpp1* deletion strain (Δ*tmus53*Δ*xpp1*) is represented schematically. Position of the *xpp1* locus on scaffold 16 is indicated at the top. Thin black arrows indicate the approximate positions of the primers used for genotype analysis via PCR (5f2, xpp1-5fwd2 and Pkr, Ppki_5rev). Black-rimmed, light gray arrow represents the *xpp1* gene; hatched boxes represent regions for homologous recombination; thick, black arrow represents the *hph* gene; gray arrow indicate the homologous recombination event; gray, dotted lines represent genomic DNA sequence; solid, black lines represent plasmid DNA sequence. (B) Correct and exclusive integration of the hygromycin cassette in the *xpp1* locus was verified by Southern Blot analysis. The obtained signals correspond to the expected fragment sizes after digestion with PstI (3,410 bp and 4,622 bp) using the *hph* coding region as probe. (C) Ectopic insertions of *xpp1* expression cassettes in the over-expression strain were determined by Southern Blot analysis using the *xpp1* coding region as probe. L, 1-kb DNA ladder; P, parent strain (QM6aΔ*tmus53*); Δ, *xpp1* deletion strain; O, *xpp1* over-expression strain. Additional file 2: Impact of Xpp1 on growth of *T. reesei*. (A) *T. reesei* QM6aΔ*tmus53* (blue) and the *xpp1* deletion strain (green) were grown in MA medium containing 1% (w/v) D-glucose (squares, solid lines) or lactose (triangles, dashed lines), or CMC (circles, dotted lines) for 18, 24, 30, 38, and 50 hours. The values provided in the figures are means from three biological experiments. Error bars indicate standard deviations. (B) *T. reesei* QM6aΔ*tmus53* (left lane) and the *xpp1* deletion strain (right lane) were pre-grown on MA medium plates containing glycerol. Equal pieces of overgrown agar were transferred to MA medium plates containing 1% (w/v) D-glucose (G) or xylan (XN) or lactose (L) or CMC. Pictures were taken after 48 hours growth at 30°C in darkness. Additional file 3: Primers used throughout this study.

## Abbreviations

Ace1/2/3: Activator of cellulases 1/2/3
CCR: carbon catabolite repression
CMC: carboxymethylcellulose
Cre1: Carbon catabolite repressor 1
DMS: dimethyl sulfate
EDTA: ethylenediaminetetraacetic acid
FAM: fluorescein amidite
GST: glutathione S-transferase
HPAEC-PAD: high-performance anion exchange chromatography with pulsed amperometric detection
ivFAST: in vivo footprinting analysis software tool
MA: Mandels–Andreotti
MEX: malt extract
qPCR: quantitative PCR
Xpp1: Xylanase promoter-binding protein 1
Xyr1: Xylanase regulator 1
