# Supplementary material for: Xpp1 regulates the expression of xylanases, but not of cellulases in Trichoderma reesei
Source: Biotechnol Biofuels. 2015 Aug 6;8:112. doi: 10.1186/s13068-015-0298-8 (PMC4526299; doi:10.1186/s13068-015-0298-8)
Supplement: Additional file 1: — Deletion and over-expression of xpp1 (A) Deletion of xpp1 in the parent strain QM6aΔtmus53 (Δtmus53) by homologous recombination with the plasmid pCD-Δxpp1 yielding an xpp1 deletion strain (Δtmus53Δxpp1) is represented schematically. Position of the xpp1 locus on scaffold 16 is indicated at the top. Thin black arrows indicate the approximate positions of the primers used for genotype analysis via PCR (5f2, xpp1-5fwd2 and Pkr, Ppki_5rev). Black-rimmed, light gray arrow represents the xpp1 gene; hatched boxes represent regions for homologous recombination; thick, black arrow represents the hph gene; gray arrow indicate the homologous recombination event; gray, dotted lines represent genomic DNA sequence; solid, black lines represent plasmid DNA sequence. (B) Correct and exclusive integration of the hygromycin cassette in the xpp1 locus was verified by Southern Blot analysis. The obtained signals correspond to the expected fragment sizes after digestion with PstI (3,410 bp and 4,622 bp) using the hph coding region as probe. (C) Ectopic insertions of xpp1 expression cassettes in the over-expression strain were determined by Southern Blot analysis using the xpp1 coding region as probe. L, 1-kb DNA ladder; P, parent strain (QM6aΔtmus53); Δ, xpp1 deletion strain; O, xpp1 over-expression strain. [file 13068_2015_298_MOESM1_ESM.pdf]

Additional file 1 – Deletion and over-expression of *xpp1*

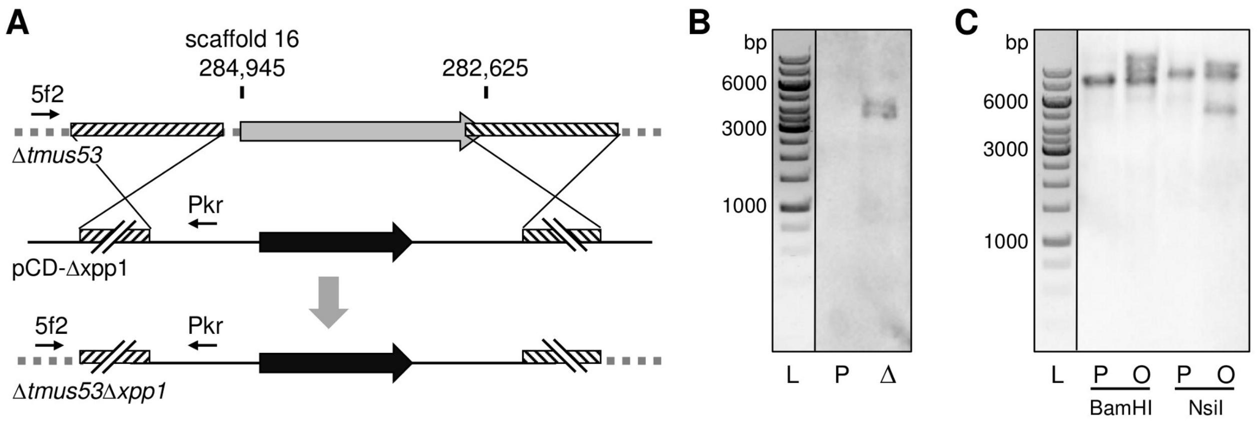

(A) Deletion of *xpp1* in the parent strain QM6aD*tmus53* (D*tmus53*) by homologous recombination with the plasmid pCD-Dxpp1 yielding an *xpp1* deletion strain (D*tmus53*D*xpp1*) is represented schematically. Position of the *xpp1* locus on scaffold 16 is indicated at the top. Thin black arrows indicate the approximate positions of the primers used for genotype analysis via PCR (5f2, xpp1-5fwd2 and Pkr, Ppki_5rev). Black-rimmed, light gray arrow represents the *xpp1* gene; hatched boxes represent regions for homologous recombination; thick, black arrow represents the *hph* gene; gray arrow indicate the homologous recombination event; gray, dotted lines represent genomic DNA sequence; solid, black lines represent plasmid DNA sequence. **(B)** Correct and exclusive integration of the hygromycin cassette in the *xpp1* locus was verified by Southern Blot analysis. The obtained signals correspond to the expected fragment sizes after digestion with PstI (3,410 bp and 4,622 bp) using the *hph* coding region as probe. **(C)** Ectopic insertions of *xpp1* expression cassettes in the over-expression strain were determined by Southern Blot analysis using the *xpp1* coding region as probe. L, 1-kb DNA ladder; P, parent strain (QM6aD*tmus53*); D, *xpp1* deletion strain; O, *xpp1* over-expression strain.
