# Supplementary material for: Xpp1 regulates the expression of xylanases, but not of cellulases in Trichoderma reesei
Source: Biotechnol Biofuels. 2015 Aug 6;8:112. doi: 10.1186/s13068-015-0298-8 (PMC4526299; doi:10.1186/s13068-015-0298-8)
Supplement: Additional file 2: — Impact of Xpp1 on growth of T. reesei. (A) T. reesei QM6aΔtmus53 (blue) and the xpp1 deletion strain (green) were grown in MA medium containing 1% (w/v) D-glucose (squares, solid lines) or lactose (triangles, dashed lines), or CMC (circles, dotted lines) for 18, 24, 30, 38, and 50 hours. The values provided in the figures are means from three biological experiments. Error bars indicate standard deviations. (B) T. reesei QM6aΔtmus53 (left lane) and the xpp1 deletion strain (right lane) were pre-grown on MA medium plates containing glycerol. Equal pieces of overgrown agar were transferred to MA medium plates containing 1% (w/v) D-glucose (G) or xylan (XN) or lactose (L) or CMC. Pictures were taken after 48 hours growth at 30°C in darkness. [file 13068_2015_298_MOESM2_ESM.pdf]

Additional file 2 – Impact of Xpp1 on growth of *T. reesei*

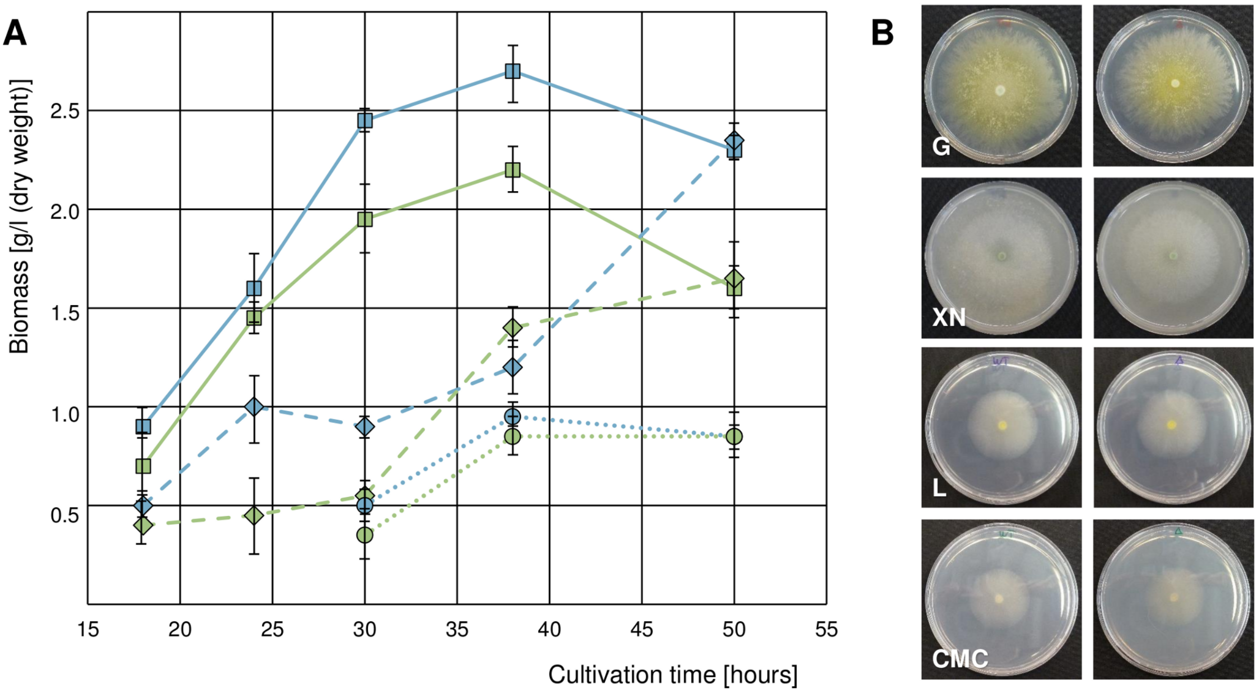

(A) *T. reesei* QM6aΔ*tmus53* (blue) and the *xpp1* deletion strain (green) were grown in MA medium containing 50 mM D-glucose (squares, solid lines), 1 % lactose (diamonds, dashed lines), or 1 % CMC (circles, dotted lines) for 18, 24, 30, 38, and 50 hours. The values provided in the figures are means from three biological experiments. Error bars indicate standard deviations. **(B)** *T. reesei* QM6aΔ*tmus53* (left lane) and the *xpp1* deletion strain (right lane) were pre-grown on MA medium plates containing glycerol. Equal pieces of overgrown agar were transferred to MA medium plates containing 50 mM D-glucose (G), 1 % xylan (XN), 1 % lactose (L), or 1 % CMC. Pictures were taken after 48 hours growth at 30°C in darkness.
