# Supplementary material for: Xpp1 regulates the expression of xylanases, but not of cellulases in Trichoderma reesei
Source: Biotechnol Biofuels. 2015 Aug 6;8:112. doi: 10.1186/s13068-015-0298-8 (PMC4526299; doi:10.1186/s13068-015-0298-8)
Supplement: Additional file 3: — Primers used throughout this study. [file 13068_2015_298_MOESM3_ESM.pdf]

Additional file 3 – Primers used throughout this study

Primer name	Primer sequence	Primer usage
xpp1-5fwd-NotI	AGAGCGGCCGCCGAGGTGGAAAAGGGTCATC	Plasmid construction
xpp1-5rev	CAGACGGAGGAGGAAGAGC	
xpp1-3fwd	ACAAGGATATCATGACGGAGC	
xpp1-3rev-NotI	AACGCGGCCGCTTCTCTTCCCGTCCCTCG	
Ppki_5fwd	AGATAACGGTGAGACTAGCGGC	
Tcbh2_rev-BcuI	ACTAGTGCTATTAACGTTTGGAAAGCCATC	
xpp1_fwd-XbaI	CACTCTAGACGCATGGCACAAGCC	
xpp1_rev-NsiI	CACATGCATCAACAGAATCCTCTCGGG	
xpp1-5fwd2	ACACTGGCCCAGAGAACTATCC	Genomic characterization
Ppki_5rev	CAGCAGCCACGACAAAGC	
RG127	GTTCCGATATATGAGATTGCCAAG	*in vivo* footrprint
RG128	GTTGATGTCTTCTTGCTTCAGC	
RG129	AGCCGTTATTCAGACAATGTATGTGCCG	
RG130	GGAGTTGTTGTGTCTTTTGGGCTTGG	
RG131	[FAM]-CCGTTATTCAGACAATGTATGTGCCGGGC	
RG132	[FAM]-GTTGTTGTGTCTTTTGGGCTTGGAGGGG	
xpp1_q2f	ATAAAGGTCGAGTCGCCGC	qPCR assays
xpp1_q2r	TGGAAGCTCTTGGCTGGTTG	
taqxyn2f	GGTCCAACTCGGGCAACTTT	
taqxyn2r	CCGAGAAGTTGATGACCTTGTTC	
xyl1-fwd	CTGTGACTATGGCAACGAAAAGGAG	
xyl1-rev	CACAGCTTGGACACGATGAAGAG	
cbh1f	GATGATGACTACGCCAACATGCTG	
cbh1r	ACGGCACCGGGTGTGG	
cbh2f	CTATGCCGGACAGTTTGTGGTG	
cbh2r	GTCAGGCTCAATAACCAGGAGG	
egl1f	CTGCAACGAGATGGATATCCTGG	
egl1r	GTAGTAGCTTTTGTAGCCGCTGC	
act1f	TGAGAGCGGTGGTATCCACG	
act1r	GGTACCACCAGACATGACAATGTTG	
sar1fw	TGGATCGTCAACTGGTTCTACGA	
sar1rv	GCATGTGTAGCAACGTGGTCTTT	
